# Children Food and Nutrition Literacy - a New Challenge in Daily Health and Life, the New Solution: Using Intervention Mapping Model Through a Mixed Methods Protocol

**DOI:** 10.25122/jml-2019-0025

**Published:** 2020

**Authors:** Mohammad Ahmadpour, Nasrin Omidvar, Aazam Doustmohammadian, Abbas Rahimiforoushani, Elham Shakibazadeh

**Affiliations:** 1. Department of Health Education and Promotion, School of Public Health, Tehran University of Medical Sciences, Tehran, Iran; 2. Department of Community Nutrition, Faculty of Nutrition Sciences and Food Technology, National Nutrition and Food Technology Research Institute, Shahid Beheshti University of Medical Sciences, Tehran, Iran; 3. Department of Nutrition Research, Faculty of Nutrition Sciences and Food Technology, National Nutrition and Food Technology Research Institute, Shahid Beheshti University of Medical Sciences, Tehran, Iran; 4. Department of Epidemiology and Biostatistics, School of Public Health, Tehran University of Medical Sciences, Tehran, Iran; 5. Department of Health Education and Promotion, School of Public Health, Tehran University of Medical Sciences, Tehran, Iran

**Keywords:** Children Food and Nutrition Literacy, New Challenge, Intervention Mapping Model, Mixed Methods Protocol

## Abstract

Food and nutrition literacy is a fundamental and new idiom among health policymakers. Improving children’s food and nutrition literacy is a fundamental task, and it requires detailed planning. The aim of this study is to design, implement, and evaluate a food and nutrition literacy promotion intervention in elementary school children based on the Intervention Mapping model.

This is a sequential study to design, implement, and evaluate a food and nutrition literacy promotion intervention in elementary school children aged 10-12 years old in Baneh city (Kurdistan, Iran). The study has three sequential phases, six steps based on the Intervention Mapping model, and four sub-studies.

The questionnaire was transformed and culturally adapted since it was previously built for the city of Tehran; the results of a population-based cross-sectional survey indicated that the score for understanding food and nutrition information of a sample of 390 students aged 10-12 was mostly moderate and low (90.3% of participants). Therefore, a qualitative study on how to fix existing barriers, and extract facilitators and the best methods of interventions for promoting food and nutrition literacy of participants was conducted. Finally, an interventional study within six months for two intervention and control groups of elementary children 10-12 years old was carried out.

The findings of this research will be used to design interventions and strategies based on needs assessment for improving students’ skills in food and nutrition literacy in all dimensions of food and nutrition literacy.

## Introduction

According to the World Health Organization (WHO), in 2017, non-communicable diseases account for 70% of deaths worldwide; according to the same report, 81% of deaths occurred in Iran due to non-communicable diseases [1]. According to the WHO report, unhealthy nutrition, with low physical activity and smoking, are common risk factors for 80% of chronic diseases [2]. The increasing prevalence of non-communicable diseases and their risk factors, especially in developing and middle and low-income countries, has been emphasized by identifying risk factors and preventing them at the individual and community levels [3-6]. Disease risk factors are often formed in childhood and are part of a human being until adolescence [7]. In Iran, statistical evidence also shows the status of high-risk behaviors in a variety of areas, such as inappropriate nutritional patterns and high consumption of ready-made foods [8, 9], low physical activity [10, 11] and the prevalence of epidemics such as overweight and obesity [11-13] does not have a favorable situation. The noteworthy point is that most of these health problems are preventable and can be avoided through health promotion programs, including promoting healthy behaviors and lifestyle changes from the early age of life [14]. The increasing prevalence of non-contagious diseases associated with nutrition, such as obesity and overweight, diabetes, hypertension and cardiovascular diseases, indicates the need to address basic health literacy and food and nutrition literacy skills [15, 16]. Evidence suggests that healthier eating practices have a positive correlation with higher food and nutrition literacy [17]. Improving food and nutrition literacy is a crucial factor in choosing the right foods and keeping the food pattern right [18, 19]; also, the review of the content of the Health Promoting Schools program in Iran shows that the dimensions of nutritional literacy and nutrition enhancement in such programs have not been adequately addressed [20]. In this study, an intervention mapping approach is used to design an intervention to improve the food and nutrition literacy of students. This model was introduced in 1998 by Bartholomew et al. [21]. One of the strengths of the intervention mapping approach is to provide a step-by-step guide to evidence-based and theory for planning and designing health promotion programs, leading to the design of coherent, comprehensive, evidence-based interventions and theories [22-25]. The success of the intervention mapping approach in designing and implementing many health promotion interventional programs such as designing educational interventions in diabetic patients [26, 27], community-centered and school-based interventions for controlling and preventing obesity [22-24], promoting healthy eating and promoting nutritional behaviors [28-31], promotion of physical activity [32-34] and the screening and cancer prevention program is visible [35, 36].

In 2013, Alizadeh et al. conducted a semi-experimental study (before and after) intending to determine the effect of education on knowledge and nutritional behavior of primary school students in Torbat Heydarieh (East of Iran) on 180 students. The results of this study showed that after the intervention, the knowledge and practice level of students in the intervention group increased significantly [37]. During 2014-2018, Doustmohammadian et al. carried out a study aimed at “designing an interventional program to improve nutritional and nutritional literacy in 10-12-year-old primary school children in Tehran city based on the intervention mapping approach”. The findings of this study suggest food and nutrition literacy as a tool for facilitating the promotion of healthy eating habits in children [38]. In 2014, Gibbs et al. conducted a study to investigate the relationship between parenteral nutrition literacy (PNL) with the Child Healthy Eating Index (CHEI) and parental body mass index. The results showed that the body mass index of children did not correlate with NLAI-P (Nutrition Literacy Assessment Instrument for Parents), but the body mass index (BMI) of parents had a significant correlation with three components of NLAI-P like food groups, consumer skills and reading nutritional tags [39]. Fleary et al. conducted a study in 2013 aimed at testing mothers-focused interventions to improve maternal health literacy and healthy lifestyle choices for children and their families. The results indicated that the increase of nutritional health literacy rates after the intervention was indicative of the usefulness of interventions in improving health literacy at least one month after the intervention [40].

The main objectives of this study are:

• Transforming and adapting the questionnaire as well as validity and reliability determination of food and nutrition literacy tools (functional, interactive and analytical) and its determinants;
• Determination of demographic characteristics, socioeconomic status and food and nutrition literacy status (functional, interactive, analytical) in the sample population;
• Determination of the status of nutritional behaviors in the sample population;
• Identifying stakeholder views on the most important sources of nutrition information and the most appropriate and feasible channel/channels for implementing food and nutrition literacy interventions in the sample population;
• Identifying stakeholder views on individual and environmental barriers and facilitators and improving food and nutrition literacy in the sample population;
• Determination and comparison of nutritional behaviors and food and nutrition literacy (functional, interactive and analytical) in the sample population before and after the intervention in both the intervention and control groups.

## Material and Methods

### Sudy Design

This is a sequential study conducted in order to design, implement, and evaluate a food and nutrition literacy promotion intervention in elementary school children aged 10-12-years old, based on the Intervention Mapping (IM) model. This mixed-methods study includes adapting the questionnaire, quantitative and qualitative methods, and an interventional period with two groups (control and intervention). The “follow-up explanation model” was adopted to explain the quantitative findings by collecting qualitative data[41].

The study has three sequential phases, six steps based on the IM model, and four sub-studies. The first phase, which needs to be measured, is the first step in the IM model and includes three sub-studies. In the first sub-study, the questionnaire was transformed and adapted since it was previously built for Tehran city (Table 1). In the second sub-study, a population-based descriptive cross-sectional study of a sample (N=390) in 10-12 years old students in terms of food and nutrition literacy and nutritional behaviors based on a native questionnaire was measured (Table 2). The collected data includes items on socio-demographic information, items on measuring food and nutrition literacy and nutritional behaviors using the proportional random multistage cluster sampling method, in Kurdistan, Iran. In the third sub-study, which is a qualitative study, facilitators extraction, methods to fix existing barriers and find the best methods of interventions in order to promote food and nutrition literacy by a purposeful sampling strategy were sought.

**Table 1:** The characteristics of the interviewees and the number of focused group discussions and interviews.

**Specialty**	**Frequency**	**Mean age (Year)**	**Gender: M(F)**	**The number of individual interviews**	**The number of focus group discussions (FGDs)**
Health education and promotion	5	45 (SD<0.05)	3(2)	5	1
Nutrition sciences	5	42 (SD<0.05)	3(2)	5	1
Pediatrician	2	47 (SD<0.05)	2	2	0
Primary school Teacher	20	46 (SD<0.05)	12(8)	20	3
Primary school Manager	10	51 (SD<0.05)	5(5)	10	1
Primary students at grades 4, 6 and 6	30 (10 per each grade)	11 (SD<0.05)	15(15)	30	5
Parents of students	15	39 (SD<0.05)	7(8)	15	1
Total number of individual interviews				Total number of focus group discussions (FGDs)	
87				12	

Data collection in the qualitative study was predominately represented by Focused Group Discussions (FGD) and individual in-depth interviews (Table 1). A qualitative content analysis approach was undertaken to develop a detailed understanding of the facilitators, understand how to fix existing barriers and extract the best methods of interventions to promote food and nutrition literacy among 10-12 years old children. In the second phase, steps 2-6 of the IM model, based on phase 1 findings, were designed. In the third phase, which includes the fourth sub-study is an interventional, executive and evaluation of interventions. The educational and skillful interventions within six months involve implementing two intervention and control groups of elementary children aged 10-12 years using the proportional random multistage cluster sampling method. After the end of the intervention, both the intervention and control groups should be compared in terms of food and nutrition literacy, nutritional behaviors, and other factors in the questionnaire in both pre- and post-intervention modes.

Generally, in the first and second sub-studies, the researcher collects and analyzes quantitative data. The second, qualitative study (sub-study 3) will be designed based on the first two sub-studies to explain the quantitative findings that need additional explanations, such as statistical differences among groups or individuals with unexpected findings. Then, the three sub-studies are connected in the interpretation stage to explain quantitative and qualitative findings [42]. The rationale for this design is that the quantitative data would provide a general understanding about food and nutrition literacy status and nutritional behaviors in the sample population and the qualitative data would explore participants’ views about the individual, interpersonal and environmental barriers and facilitators of food and nutrition literacy and nutritional behaviors in the sample population (Table 3).

**Table 2:** Demographic characteristics and the score of understanding food and nutrition information for students aged 10 - 12 years derived from sub-study 2.

			**Frequency**	**Percent**
**Gender**	Male		195	50
	Female		195	50
**Total No**	390			100
**Educational Grade**	4		130	33.3
	5		130	33.3
	6		130	33.3
**Total No**	390			100
**Age**	10y	Mean=11y (SD<0.05)	130	33.3
	11y		130	33.3
	12y		130	33.3
**Total No**	390			100
**Quartet Height**	Quartile 1		114	29.2
	Quartile 1		179	45.9
	Quartile 1		97	24.9
**Total No**	390			100
**Quartet Weight**	Quartile 1		97	24.9
	Quartile 1		193	49.5
	Quartile 1		100	25.6
**Total No**	390			100
**The score of understanding of food and nutrition information**	Low		220	56.4
	Moderate		132	33.8
	High		38	9.7
**Total No**	390			100

**Table 3:** The protocol for improving food and nutrition literacy (FNLIT) of Children aged 10-12 years based on the IM model. [The study framework derived from (Creswell et al., 2011)(44)].

**Phase**	**Procedure**	**Product**
**Phase 1 (Step1 of the IM model)**		
Sub-study 1 (Adapting the questionnaire)	Test-Retest + CVR & CVI + Cronbach’s Alpha	Native Questionnaire
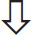		
Sub-study 2 (Check existing status using a cross-sectional survey)	Descriptive cross-sectional study) (n=384) + SPSS Software	Descriptive and Analytic Statistics Regression
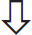		
Sub-study 3 (Qualitative Study)	- Individual in-depth Interviews - Content Analysis	Text data (Extract barriers and facilitators for improving food and nutrition literacy)
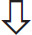		
**Phase 2**		
Integration of Quantitative and Qualitative Findings	Interpreting and explaining the results of quantitative and qualitative studies	Designing interventions based on native cultural, social and economic considerations
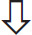		
Design steps 2-6 of the Intervention Mapping Model based on Phase 1 and 2 findings	- Draw a matrix of change goals - Methods determination based on the theory - Design and select the content of the program - Design adoption and implementation - Design program evaluation	Map of the Implementation of the Interventional Study
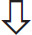		
**Phase 3**		
Sub-study 4 (Interventional study) = (Execution of the designed steps - 2-6 of the IM Model)	- Identification of intervention and control groups - Pre-test in intervention and control groups - Educational and skillful interventions in intervention group based on the steps of the IM model - Post-test in intervention and control groups	Comparison of intervention and control groups based on nutrition and nutrition literacy and nutritional behaviors in both pre- and post-intervention modes.

## Results

### Sub-study 1: (Adapting the questionnaire)

In this sub-study, the questionnaire was transformed and culturally adapted since it was previously built for the city of Tehran. The face and content validity were examined, and then the Test-Retest method was used to measure its reliability. The main questionnaire items were made by at least five people in each field, totally by 20 people, including Health Promoters and Nutritionists, Pediatricians, School Directors, Teachers, and School Health Instructors with at least a Bachelor’s degree (Table1). The face and content validity of the new questions was calculated, and then the test-retest method was used to measure its reliability. Since the main questionnaire consists of 46 questions and each question was completed at least by three students, then the questionnaires were filled in by at least 138 students before and after ten days. After that, the internal correlation coefficient and Cronbach’s Alpha of the questions were also calculated. Two relative content validity coefficients (CVR), and content validity index (CVI) were used to evaluate content validity quantitatively.

### Sub-study 2: (Descriptive cross-sectional study)

In this sub-study, a population-based cross-sectional survey of a sample of 10-12 years old students in terms of food and nutrition literacy and nutritional behaviors based on a native questionnaire was measured. Due to the lack of specific information in the community of students regarding the ratio of students who have or do not have food and nutrition literacy, this ratio was considered to be 50%, and the number of samples was determined so that a 95% confidence interval and a maximum 5% error could be attributed to this ratio in the community of students; the proportion of elementary students in grades 4, 5 and 6 was calculated equally. The total sample included 390 students. The results from the second sub-study indicate that the score for understanding food and nutrition information was mostly moderate and low (90.3% of participants). More information is provided in Table 2.

### Sub-study 3: (Qualitative Study)

In the third sub-study, which is a qualitative study on facilitators extraction, methods to fix existing barriers and find the best methods of interventions in order to promote food and nutrition literacy by a purposeful sampling strategy were sought (Table1).

Data collection in the qualitative study was represented predominately by Focused Group Discussions (FGD), and individual in-depth interviews using a pre-established interview guide. Before any interviews, recording equipment should be provided. The data will be recorded through tape recording and note-taking. Interviews were continued until data saturation, and no new themes related to the phenomenon under study emerged. The content analysis method was used. The codes were created and grouped based on their similarity into several categories to reveal the interviewees’ comments and perceptions on extracting facilitators, how to fix existing barriers, and to extract the best methods of interventions to promote food and nutrition literacy. Peer and member checks were performed to assess the trustworthiness of the data [43]. Qualitative data were managed using the MAXQDA10 software.

### Sub-study 4: (Interventional study)

Evaluation of interventions regarding the educational and skillful interventions within six months involves implementing two groups (intervention and control) of elementary children aged 10-12 years using the proportional random multistage cluster sampling method. After six months of educational and skillful interventions in the intervention group, the data were collected as described in the Sub-study 2 section. Also, both the intervention and control groups were compared based on each item of all three demographics, food and nutrition literacy and nutritional behaviors questionnaires. After the end of the intervention, both groups were compared based on the seven dimensions of food and nutrition literacy questionnaires and nutritional behaviors questionnaire using the SPSS 19 software.

### Data Integration: (Integration Quantitative and Qualitative Findings)

Because the study is designed as a sequence based on the intervention mapping model, quantitative (Sub-study2) and qualitative (Sub-study3) study data will be analyzed separately because the qualitative results depend on the quantitative results [45]. Then, the findings of both quantitative and qualitative studies are used to design the steps of the intervention mapping model to use them in an interventional study (Sub-study 4).

## Discussion

Improving food and nutrition literacy is a key factor in choosing the right foods and keeping the pattern of food right [18]. The results of the second sub-study indicate that the score for understanding food and nutrition information was mostly moderate and low (90.3% of participants) compared to a cross-sectional study conducted by Doustmohammadian et al. [38] in Tehran city in 2014, in which the participants were similar to the participants in this study, except for their ethnicity and place of residence, weaker results were obtained. In their study, 49.1% of participants had a moderate and upward level of nutrition. This shows that the level of food and nutrition literacy of students in this area is more inappropriate than Tehran city’s students and requires thorough and comprehensive planning to improve it, which is one of the goals of this study. In another cross-sectional study which was conducted by V.Y Feyzabadi et al. in 2017 on 1320 female students in Kerman, Iran, the status of nutritional knowledge and unhealthy nutrition behaviors was assessed through a questionnaire. The results showed that lower nutritional knowledge (PRR = 0.96; 95% CI: 0.91-0.99) was associated with higher weekly unhealthy snaking. The results also indicate that nutritional knowledge and unhealthy nutrition behaviors are affected by individual, social, and cultural factors, and it is necessary to pay attention to all aspects in order to improve nutrition knowledge and unhealthy nutrition behaviors [19]. In line with the implementation of the fourth sub-study, an interventional study conducted to compare food and nutrition literacy between the two intervention and control groups by Pirzadeh et al. in 2011, examined the effect of training on nutritional behavior based on the Beliefs, Attitudes, Subjective Norms and Enabling Factors (BASNEF) model on female students. The results indicated that the mean scores of knowledge and components related to the BASNEF model were significantly different between the two groups. While nutritional performance improved, including receiving different food groups (dairy products, meat and protein, fruits and vegetables in the intervention group) [46]. In another study, Alizadeh et al. conducted in 2013 a semi-experimental study (before and after) with the aim of determining the effect of education on knowledge and nutritional behavior of primary school students in Torbat Heydarieh (East of Iran) on 180 students. The results of this study showed that after the intervention, the knowledge and practice level of students in the intervention group increased significantly [37]. In another intervention study conducted by Azam Fathi et al. in 2016 in Qom city, Iran, 88 sixth- grade girls were selected in two intervention and control groups using the multistage random sampling method. The results showed that before the intervention, the mean score of knowledge and practice of the intervention group was 96.12 and 18.61, respectively, which changed to 110 and 68.22 after the intervention. The results showed that there was no significant difference in the mean score of nutritional knowledge before the intervention between the two groups (P>0.05). After the intervention, the scores of all changes and satisfactory feeding behaviors in the experimental group significantly increased (P<0.05) [47]. Several nutritional studies have used the IM model, including promoting healthy eating and promoting nutritional behaviors [28-31]. Therefore, the main strength of this study is that it uses both quantitative and qualitative studies data based on the IM model to design an interventional study. The combination of quantitative and qualitative methods allows us to understand the interconnection, complexity of social norms of human beings, and social determinants of health to promote food and nutrition literacy and improve nutritional behaviors [48, 49]. In addition, this study explores factors related to food and nutrition literacy promotion and improving nutritional behaviors to develop effective strategies and interventions. The study results are expected to help health policymakers, the health promotion department of the Ministry of Health and the Ministry of education in due course.

## Acknowledgments

Tehran University of Medical Sciences supported this research as a Ph.D. student dissertation (Dissertation code: 9511108004).

## Conflict of Interest

The authors confirm that there are no conflicts of interest.

## Ethics

IRCT Code: 32094- 14.07.2018. The protocol for this study was approved by the Ethics Committee of Tehran University of Medical Sciences. Written informed consent was obtained from all participants before the study. This research was evaluated by the School of Public Health and Allied Medical Sciences- Tehran University of Medical Sciences (Approval ID: IR.TUMS.SPH.REC.1397.031 - Approval Date: 2018/06/13).
